# Physical characterization of a new CT iterative reconstruction method operating in sinogram space

**DOI:** 10.1120/jacmp.v14i4.4347

**Published:** 2013-07-08

**Authors:** Caterina Ghetti, Francesca Palleri, Giulio Serreli, Ornella Ortenzia, Livia Ruffini

**Affiliations:** ^1^ Servizio di Fisica Sanitaria Azienda Ospedaliero‐Universitaria Parma Italy; ^2^ Dipartimento di Diagnostica per Immagini Azienda Ospedaliero‐Universitaria Parma Italy

**Keywords:** iterative reconstruction, image quality, computed tomography

## Abstract

Recently a new iterative reconstruction algorithm named Sinogram Affirmed Iterative Reconstruction (SAFIRE) has been released by Siemens. This algorithm works in the raw data domain with noise reduction as main purpose, providing five different strengths. In this study, the effect of SAFIRE on image quality has been investigated using selected phantoms and a comparison with standard filtered back projection (FBP) has been carried out. The following quantitative parameters have been evaluated: image noise, impact of different reconstruction kernels on noise reduction, noise power spectrum (NPS), contrast‐to‐noise ratio (CNR), spatial resolution, and linearity and accuracy of CT numbers. The influence of strengths on image quality parameters has also been examined. Results show that image noise reduction is independent of reconstruction kernel and strongly related to the strength of SAFIRE applied. The peak of NPS curve for SAFIRE reconstructions is shifted towards low frequencies; this effect is more marked at higher levels of strength. Contrast‐to‐noise ratio is always improved in SAFIRE reconstruction and increases with higher strength. At different dose levels SAFIRE preserves CT number accuracy, linearity, and spatial resolution, both in transversal and coronal planes. These results confirm that SAFIRE allows for image noise reduction with preserved image quality. First clinical data to validate this phantom analysis and confirm that commercially available iterative algorithms can play an effective role in dose containment.

PACS number: 87.57.Q

## I. INTRODUCTION

The use of iterative reconstruction algorithms in computed tomography (CT) has become a crucial issue for dose reduction in CT examinations. The main advantage of iterative algorithms as opposed to filtered back projection (FBP) is the incorporation of physical models, which allow for CT studies at reduced doses with preserved image quality and low levels of image noise.^1^, ^2^, ^3^, ^4^, ^5^, ^6^, ^7^, ^8^, ^9^ The most important iterative reconstruction methods and the solutions introduced by CT manufacturers have been recently reviewed.^(10)^

The latest reconstruction algorithm introduced by Siemens is Sinogram Affirmed Iterative Reconstruction (SAFIRE). It is FDA‐approved and it is considered innovative compared to previous algorithm of the family, Iterative Reconstruction in Image Space (IRIS),^(11)^ as it works not only in image space but also in raw data domain. First, an anisotropic noise model is applied to images reconstructed with FBP in order to reduce the variance of the signal. After each iteration, data are reprojected in sinogram space to validate (or affirm) the images with measurement data, and the detected deviations are corrected, yielding an updated image.^(12)^

Previous clinical studies exploring SAFIRE reconstruction have measured parameters such as contrast‐to‐noise ratio (CNR) and signal‐to‐noise ratio (SNR), and provided a subjective assessment of image quality. Most of these studies report an image noise reduction without loss of diagnostic information, and consistent dose reduction.^12^, ^13^, ^14^, ^15^, ^16^, ^17^, ^18^, ^19^, ^20^, ^21^, ^22^

Neverthless, an accurate quantitative characterization of SAFIRE reconstruction is not available in the literature. The aim of this study is thus to evaluate the SAFIRE algorithm using image quality parameters measured on phantoms in order to describe the effect of iterative reconstruction with objective metrics.

Noise, noise power spectrum (NPS), CNR, kernel impact on noise reduction, linearity and accuracy of CT numbers, and both transverse and coronal spatial resolution have been investigated using dedicated phantoms, and results have been compared to traditional FBP.

## II. MATERIALS AND METHODS

Measurements were performed on a SOMATOM Definition Flash CT scanner (Siemens Healthcare, Malvern, PA), a dual‐source system equipped with two 64 row detector arrays.^(23)^

The SAFIRE algorithm (Siemens Healthcare) is available in all helical protocols and can be selected during the reconstruction stage of an imaging procedure. A wide selection of reconstruction kernels is available on Siemens CT scanners and most of them have a correspondence with the conventional filters used in FBP. A FBP soft filter such as B31S, for example, corresponds to I31S kernel in iterative reconstruction.

It is possible to choose between five different strengths of SAFIRE (S1–S5), ranging between level one and five. The number of iterations loops employed is a hidden parameter and it cannot be modified by the user. The reconstruction time is around 20 images/sec.

The effect of SAFIRE on image quality was investigated through the imaging of several phantoms: a water phantom, a Catphan 600 phantom (The Phantom Laboratory, Salem, NY),^(24)^ and a 3D spatial resolution phantom (QRM, Möhrendorf, Germany).^(25)^ A comparison between FBP and SAFIRE reconstructions of the same datasets has been performed. In Table 1, the acquisition/reconstruction parameters used are reported.

The water phantom used consisted of a 30 cm diameter acrylic cylinder phantom filled with water. It was acquired with a thorax routine protocol at 120 kVp changing the tube current in order to explore a wide range of CT dose index (CTDIvol), from 3.4 to 20.2 mGy. CTDIvol values displayed on the CT workstation were verified by direct measurements in a 32 cm diameter dedicated polymethylmethacrlyate (PMMA) phantom with a calibrated Victoreen NERO mAx 8000 equipment and a pencil ionization chamber model 6000–100 (Victoreen Instrument Co., Cleveland, OH).^(26)^

Images were processed using a conventional FBP kernel (B31s) and the corresponding SAFIRE filter I31s at all strengths. Noise reduction was evaluated in a circular region of interest ROI (100×100pixels) positioned at the phantom centre. Results were expressed as standard deviation (SD) of CT numbers.

With a fixed strength of SAFIRE at S3 and CTDIvol at 13.4 mGy, different kernels (B36s, B40s, B70s versus I36s, I40s, I70h) were selected to evaluate a possible difference in noise reduction of SAFIRE due to the filter applied.

However, the evaluation of noise properties of an image using SD metric is not exhaustive because the image appearance depends also on the noise distribution in frequencies, described by noise power spectrum NPS.^(28)^ For this reason, and according to the current literature,^1^, ^27^, ^28^, ^29^ this parameter was calculated for the images acquired with water phantom and reconstructed with FBP B40s and SAFIRE I40s (S1–S5) kernel at 13.4 mGy of CTDIvol. The 2D NPS($f_{x},f_{y}$) was computed over 10 images on an area of 20 cm^2^, containing 12 overlapping ROISs of 128×128pixels. One‐dimensional NPS curve was also determined by averaging data along $f_{x}$ and $f_{y}$ directions in frequency domain.^(27)^

**Table 1:** Details on protocols and phantoms used in the study

*Physical Parameter Investigated*	*Scan Parameters*	*Reconstruction Parameters*	*Phantom Used*
Water mean CT value and standard deviation versus dose and SAFIRE strength (Fig. 1)	120 kV, 300/200/100/50 mAs, 1.0 sec rotation time, 128×0.6mm collimation, 2 mm slice thickness, pitch 1.0	FBP:B31s SAFIRE: I31s S1, S2, S3, S4, S5	30 cm diameter water‐filled phantom
Water standard deviation versus different reconstruction kernels (Table 2)	120 kV, 200 mAs, 1.0 sec rotation time, 128×0.6mm collimation, 2 mm slice thickness, pitch 1.0	FBP:B31s, B36s, B40s, B70s SAFIRE: I31s, I36s, I40s, I70h S3	30 cm diameter water‐filled phantom
Noise power spectrum NPS (Fig. 2)	120 kV, 200 mAs, 1.0 sec rotation time, 128×0.6mm collimation, 2 mm slice thickness, pitch 1.0	FBP: B40s SAFIRE: I40s S1, S2, S3, S4, S5	30 cm diameter water‐filled phantom
CT number linearity and CT number accuracy (Table 3)	120 kV, 200 mAs, 1.0 sec rotation time, 128×0.6mm collimation, 2 mm slice thickness, pitch 1.0	FBP:B31s SAFIRE: I31s S1, S3, S5	Catphan 600, sensitometry module
Low contrast resolution (Fig. 3, Table 4)	120 kV, 300/200/100 mAs, 1.0 sec rotation time, 128×0.6mm collimation, 2 mm slice thickness, pitch 1.0	FBP : B31s SAFIRE: I31s S1, S2, S3	Catphan 600, low‐contrast resolution module
Transverse spatial resolution and modulation transfer function MTF (Fig. 4)	120 kV, 200/50 mAs, 1.0 sec rotation time, 128×0.6mm collimation, 2 mm slice thickness, pitch 1.0	FBP: B70s SAFIRE: I70h S1, S3, S5	Catphan 600, high‐resolution module
Coronal spatial resolution (Fig. 5)	120 kV, 500 mAs, 1.0 sec rotation time, 128×0.6mm collimation, pitch 1.0	FBP: B46f SAFIRE: I46f S5 MPR: 1 mm recon thickness, 0.1 mm image increment	3D spatial resolution phantom

Another set of measurements was performed using a Catphan 600 phantom. The sensitometry module was scanned to verify that SAFIRE reconstruction does not affect CT number accuracy and linearity.

The module contained different targets made of teflon, delrin, acrylic, polystyrene, low‐density polyethylene (LDPE), and polymethylpentene (PMP). It was scanned at 120 kVp and resulted in a CTDIvol^(26)^ of 13.4 mGy. Images were reconstructed with traditional FBP (B31s) and with three different levels of SAFIRE strength (I31s S1, S3, S5).

Since noise reduction is supposed to improve low‐contrast detectability, the low‐contrast Catphan 600 module was scanned to quantify this effect. It is composed by target discs arranged in three groups with nominal contrast of 0.3%, 0.5%, and 1.0%, and decreasing diameters from 15 mm to 2 mm. Images were acquired at 120 kVp with three different dose levels (CTDIvol of 20.2, 13.4, and 6.7 mGy) and then reconstructed with FBP (B31s) and with SAFIRE (I31s S1, S3, and S5).

The CNR was evaluated by subtracting the mean CT value measured in the 15 mm diameter detail at 1% nominal contrast from the mean CT number measured nearby in phantom background. Results are divided by the standard deviation of the pixel values of the phantom background.^(30)^ The CNR calculation was repeated for images reconstructed with FBP and SAFIRE (S1, S3, and S5) at three different dose levels.

The effect of the SAFIRE algorithm on spatial resolution in the transverse and coronal planes was explored using the Catphan 600 transverse spatial resolution module and the 3D spatial resolution phantom.

Catphan 600 transverse spatial resolution module is comprised of bar patterns with different spatial frequencies ranging between 1 and 21 lp/cm. In the same module, two bead point sources are available to calculate the line spread function (LSF) in vertical and horizontal direction and then, applying to it a one‐dimensional Fourier transform, the modulation transfer function (MTF) of the system^(31)^ was determined.

The bar patterns were acquired at 120 kVp with two dose levels (CTDIvol of 13.43 and 3.4 mGy). Images were then reconstructed with a high‐resolution kernel using FBP (B70s) and SAFIRE (I70h) at strengths S1, S3, and S5. To obtain the image of the bead sources, an acquisition was performed using 120 kVp at CTDIvol of 20.2 mGy and a reconstruction FOV of 50 mm with a B70s kernel for FBP and a I70h strength S3 for SAFIRE.

The 3D spatial resolution phantom consists of circular holes of varying diameter from 4.0 mm down to 0.4 mm both in the x–y plane and along the z‐axis. It was scanned at 120 kVp with a CTDIvol of 33.5 mGy, and reconstructed in the transverse and coronal plane with FBP (B46s) and with SAFIRE (I46s S5).

All images were evaluated with ImageJ 1.43u software (U. S. National Institutes of Health, Bethesda, MD).

## III. RESULTS & DISCUSSION

A good agreement, within 2.5%, between displayed and measured values of CTDIvol was obtained, suggesting that subsequent clinically calculated volume doses were representative of actual doses.

**Figure 1:**
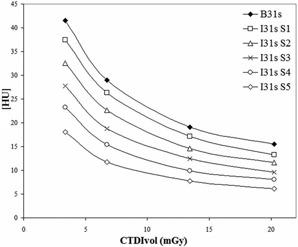
Image noise as standard deviation (SD) obtained in a 30 cm diameter water‐filled phantom as a function of CTDIvol using FBP algorithm (B31s) and SAFIRE reconstruction at different levels of strength (I31s S1, S2, S3, S4, S5).

Figure 1 shows image noise (represented as SD) as a function of CTDIvol for FBP and for different strengths of SAFIRE. The noise reduction of iterative reconstruction increases with the SAFIRE strength applied in a proportional way. For example, for a CTDIvol of 13.43 mGy, there is a decreasing of SD that is −10%,−23%,−35%,−48%, and −59% for SAFIRE S1, S2, S3, S4, and S5, respectively. The noise reduction percentage is independent of dose if the strength of SAFIRE is fixed. Standard deviation reduces as the inverse square root of CTDIvol as expected for FBP, which means that the Poisson distribution of noise is conserved in this iterative reconstruction method.

In Table 2, the percentage of noise reduction that can be obtained with SAFIRE S3 using different reconstruction kernels is reported. The standard deviation obtained with traditional FBP filter is compared to the SD obtained with its iterative homologous kernel (B31s vs. I31s, B36s vs. I36s, B40s vs. I40s, B70s vs. I70s). There is no evidence of a significant difference between different filters in the SAFIRE outcomes.

**Figure 2:**
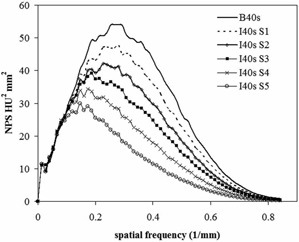
NPS curves for FBP (B40s) and SAFIRE (I40s strengths S1‐S5) reconstruction.

Figure 2 shows the frequency distribution of noise expressed by NPS, for traditional FBP (B40s) and for SAFIRE S1, S2, S3, S4, S5 (I40s). The area under the NPS curve represents the cumulative amount of noise. It is evident that noise decreases as the strength of SAFIRE increases. The shape of NPS curve obtained with FBP is comparable to data available in the literature^28^, ^29^ for B40 reconstruction kernel.

It can be also noticed that shapes of SAFIRE NPS curves are different from FBP; peaks are shifted towards low frequencies and this effect is more marked for strengths S4 and S5.

Results of analysis on the Catphan 600 sensitometry module are reported in Table 3. The mean CT values measured over seven test objects of different electron density remain the same using SAFIRE at every strength. There are no considerable differences from values obtained with FBP. The linear correlation coefficient between CT number and nominal targets relative electron density is equal to 0.996 in all configurations. The differences between nominal insert values and measured CT numbers obtained with both reconstruction methods are imputable to a different acquisition protocol and scanner model, as reported in the Catphan 600 User Manual.^(24)^

**Table 2:** Percentage of noise reduction (SD metric) obtained with SAFIRE reconstruction at strength S3 versus traditional FBP for different kernels

*Kernel*	*SD (HU)*	$\left({\text{SD}}_{\text{SAFIRE}}-{\text{SD}}_{\text{FBP}})/{\text{SD}}_{\text{FBP}}(\%\right)$
FBP B31s SAFIRE I31s S3	19.09 12.44	−35%
FBP B36s SAFIRE I36s S3	23.46 14.96	−36%
FBP B40s SAFIRE I40s S3	22.9 14.56	−36%
FBP B70s SAFIRE I70h S3	89.05 55.03	−38%

**Figure 3:**
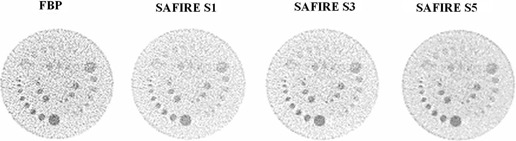
Low‐contrast module of Catphan 600, acquired at 20.2 mGy of CTDIvol and reconstructed with FBP and SAFIRE (window width ww=80,window center wc=80).

A selection of images acquired with the low‐contrast module of the Catphan 600 is shown in Fig. 3. Detail edges are sharper with less background noise using SAFIRE. Image texture changes increase with SAFIRE strength, with an overall image quality improvement. Contrast‐to‐noise ratio evaluations are showed in Table 4. CNR is always greater for SAFIRE and it increases with the strength of SAFIRE applied.

**Figure 4:**
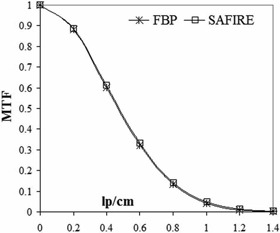
MTF computed for FBP (B70s) and SAFIRE (I70h strength S3) reconstruction.

Figure 4 shows the MTF curves calculated using the image of Catphan 600 with bead source reconstructed with traditional FBP and SAFIRE S3. The two curves are superimposed with no improvement in spatial resolution using the iterative algorithm. The same result was obtained with high‐resolution module of Catphan 600 in terms of bar pattern detection, equally for both reconstructions.

**Table 3:** Data obtained with FBP and SAFIRE reconstruction in sensitometry module of Catphan 600

	*Air*	*PMP*	*LDPE*	*Polystyrene*	*Acrylic*	*Delrin*	*Teflon*
Nominal CT Number	‐1000	‐200	‐100	‐35	120	340	990
FBP B31s	−960.8	−175.8	−87.5	−29.1	118.8	319.2	904.2
SAFIRE I31s S1	−960.9	−175.0	−88.0	−28.8	118.9	318.6	904.5
SAFIRE I31s S3	−960.7	−174.3	−88.5	−29.4	118.8	318.6	903.7
SAFIRE I31a S5	−960.5	−174.9	−88.2	−29.3	118.6	318.6	904.7

**Table 4:** CNR evaluated in low‐contrast module of Catphan 600 for different acquisition doses and reconstructions

*Reconstruction*	*CNR at 20.2 mGy of CTDIvol*	*CNR at 13.4 mGy of CTDIvol*	*CNR at 6.7 mGy of CTDIvol*
B31s FBP	2.48	1.81	1.75
SAFIRE I131s S1	2.85	2.17	1.83
SAFIRE I131s S3	3.60	2.80	2.62
SAFIRE I131s S5	5.83	4.35	3.37

**Figure 5:**
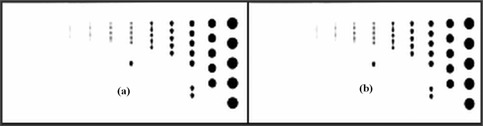
Coronal images of the 3D spatial resolution phantom: (a) FBP reconstruction, and (b) SAFIRE S5 reconstruction (ww=1100,wc=100).

Multiplanar reconstructions (MPRs) obtained in the coronal plane for FBP and SAFIRE S5 using the 3D spatial resolution phantom are presented in Fig. 5. Also, in this case, there is no difference in detail visualization between standard and iterative reconstruction.

## IV. CONCLUSIONS

The features of SAFIRE, the new iterative algorithm available on Siemens CT scanners, were explored using a phantom‐based approach. Spatial resolution is preserved by SAFIRE both in transverse and coronal planes, even at low‐dose levels. Accuracy and linearity in CT number are not affected by iterative reconstruction. SAFIRE is able to decrease image noise with a reduction up to 60%. This effect is independent from the kernel, but strongly related to the strength of SAFIRE applied. As a direct consequence, low‐contrast detectability (in term of CNR) is improved by SAFIRE, suggesting that a consistent dose reduction can be performed in clinical protocols using this iterative reconstruction method.

Another aspect examined is image texture in term of NPS; with SAFIRE strength of S4 and S5, the peak of the NPS curve is shifted towards low frequencies. This effect is coupled with a blotchy image quality impression. The fact that the user has the possibility to change different strengths in SAFIRE application is especially important. In this way, a good compromise can be reached between dose reduction and a familiar image appearance.

First clinical data^12^, ^13^, ^14^, ^15^, ^16^, ^17^, ^18^, ^19^, ^20^, ^21^, ^22^ validate this phantom analysis and confirm the role of SAFIRE in dose reduction in some anatomical regions such as lung, liver, heart, and abdomen, and in pediatric and obese patients.
